# *QuickStats:* Percentage* of Adults Aged 18–64 Years Who Had a Dental Visit in the Past 12 Months,^†^ by Dental Insurance^§^ and Year — National Health Interview Survey, United States, 2019–2020^¶^

**DOI:** 10.15585/mmwr.mm7116a3

**Published:** 2022-04-22

**Figure:**
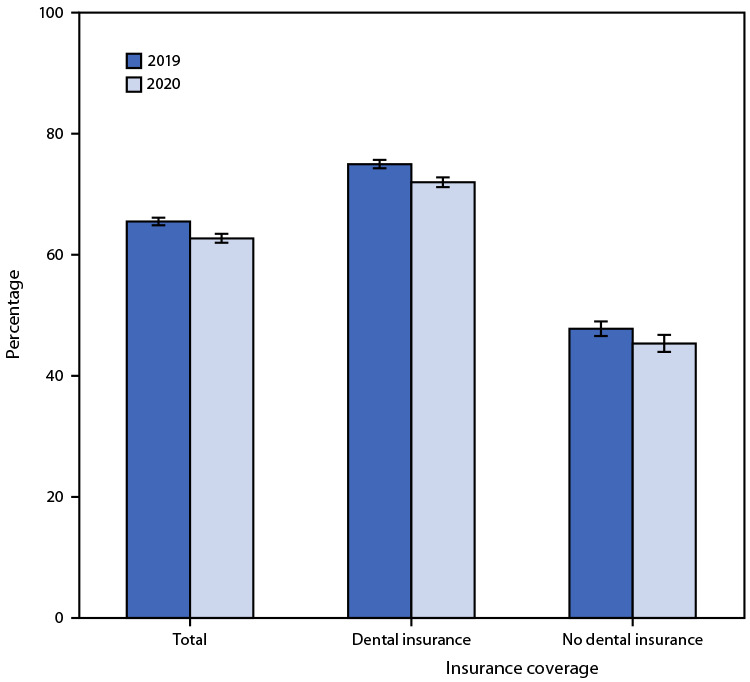
The percentage of adults aged 18−64 years who had a dental visit during the past 12 months decreased from 65.5% in 2019 to 62.7% in 2020. From 2019 to 2020, the percentage of adults aged 18−64 years who had a dental visit during the past 12 months decreased for those with dental insurance (75.0% to 72.0%) and those without dental insurance (47.8% to 45.4%). In both 2019 and 2020, adults with dental insurance were more likely to have a dental visit than those without dental insurance.

**Source:** National Center for Health Statistics, National Health Interview Survey, 2019 and 2020. https://www.cdc.gov/nchs/nhis.htm

For more information on this topic, CDC recommends the following link: https://www.cdc.gov/oralhealth/oral_health_disparities/index.htm

